# Thermal Degradation Characteristics of Styrene-Butadiene-Styrene Copolymer Asphalt Binder Filled with an Inorganic Flame-Retarding Agent

**DOI:** 10.3390/polym14183761

**Published:** 2022-09-08

**Authors:** Xiaobing Chen, Zhenyu Ma, Jianguang Zhou, Juntian Wang, Xiaorui Zhang, Ronglong Zhao, Jinhu Tong

**Affiliations:** 1 School of Transportation, Southeast University, Nanjing 211189, China; 2 Architects and Engineers Co., Ltd. of Southeast University, Nanjing 210096, China; 3 Suzhou Transport Investment, Planning, Design and Management Co., Ltd., Suzhou 215006, China; 4 Hangzhou Transportation Development Support Center, Hangzhou 310030, China

**Keywords:** flame-retarded asphalt binder, flame-retardant performance, thermal degradation characteristics, rheological properties, STA, FTIR, DSR

## Abstract

Asphalt binder is a complex mixture of dark brown polymers composed of hydrocarbons with generally poor fire resistance. To improve its flame retardancy when used in tunnel asphalt pavements, a new inorganic flame-retardant filler (FR) containing magnesium hydroxide, aluminum hydroxide, inorganic phosphate, and melamine salt was explored. Thereafter, limiting oxygen index (LOI) and smoke suppression tests for the flame-retarded asphalt binder (FRA) mastics mixed with FR and styrene-butadiene-styrene (SBS) copolymer asphalt binder were conducted. Thermogravimetric (TG) and differential scanning calorimetry (DSC) curves for the FRA were correspondingly generated. Based on the TG data, the reaction function *g*(*α*), apparent activation energy *E*_a_, and pre-exponential factor *A* were quantitatively evaluated using kinetic analysis. In addition, a Fourier transform infrared spectrometry (FTIR) test was utilized to assess the effects of the presence of FR on the chemical composition of the asphalt binder. Dynamic shear rheometer (DSR) tests were also performed to evaluate the rheological behavior of FRA. Results show that the presence of the FR significantly reduced the LOI and improved the smoke suppression during combustion of the asphalt binder mastics. The presence of FR was found to increase the *E*_a_ and the complexity of the combustion reaction, thereby improving the flame retardancy of the asphalt binder. FTIR analysis indicated that the presence of FR did not induce any strong chemical reactions to significantly impact or alter the functional groups of the asphalt binder. Furthermore, it was also observed that the rutting parameter and critical failure temperature of FRA increased with the addition of FR due to the stiffening effect of the solid FR particles.

## 1. Introduction

Asphalt pavement is one of the preferred structures for use in urban tunnels partly because of its good skid-resistance, smoothness, quietness, driving comfort and easy maintainability [1,2]. However, due to the narrow and closed space as well as the frequent occurrence of traffic congestion, tunnel fires can have disastrous consequences. The asphalt pavement can become a secondary source of fuel for the fire. Asphalt binder is a complex mixture of polymers composed of various hydrocarbons and, thus, easy to combust because of its large volatile content at high temperatures [3,4,5]. During the asphalt pyrolysis process, a large amount of toxic smoke is released, which can seriously affect evacuation and rescue operations [6,7,8,9]. Therefore, it is necessary to study the preparation of flame-retardant asphalt binder (FRA) including its thermal degradation characteristics to permit improvements in the infrastructural resilience of urban tunnels with respect to withstanding and absorbing fire-generated energy [10,11].

Styrene-butadiene-styrene (SBS) copolymer asphalt binder is one of the most widely used modified asphalt binders and can be an important raw material for preparing FRA [12]. The thermal degradation of SBS copolymer is a self-catalyzed reaction mainly occurring in butadiene blocks, and is comprised of four steps, namely: initiation of the chains by radical, growth and decomposition of the polymer chain, formation of the anhydride from dehydrated carbonyl, and annihilation of the active centers [13]. When SBS copolymer is mixed with the base asphalt binder, its thermal degradation will have an interaction with the asphalt substrate. The decomposition of SBS has the effect of inhibiting polymerization of the molecules in asphaltene; the low molecular weight substrate in the asphalt will enhance the decomposition of SBS polymer [12]. The pyrolysis processes of the SBS copolymer asphalt binder include oil content release stage, resin pyrolysis stage, and asphaltene and char combustion stage [13,14].

A common method for improving the thermal degradation behavior and reducing the flammability for asphalt binder is to blend the asphalt with organic or inorganic flame retardants to produce FRA [15,16,17]. The action of flame retardants might include the interruption of the heat exchange, promotion of condensed phase char formation, gaseous phase interruption of the combustion processes, and a cooling-dilution effect [2]. Halogenated flame retardants, which are organic additive flame-retarding agents, have been proved to have good flame-retarding effects in asphalt binders and are widely used in tunnel asphalt pavements. The halogen flame retardants take effect by decomposing and producing non-combustible hydrogen halide gas to cover the surface of the asphalt binder, and they are also effective for scavenging radicals in the reaction [8]. However, due to the toxicity and corrosion of the decomposition products and the complexity of the construction process, halogenated flame retardants and some other flame retardants such as antimony trioxide and zinc borate have been banned from use in many areas. As a result, halogen-free inorganic fillers have attracted increasing interest to serve as a substitute [8,18].

In recent years, hydroxides have become extensively used as replacements for halogen-containing flame retardants. The flame retardancy of FRA with hydroxide is realized by the endothermic effect in the hydroxide decomposition process and the formation of a film of oxide produced from this process [19]. As reported in the literature, asphalt binder blended with metal hydroxide has good flame retardancy and smoke suppression because the decomposition temperature of the metal hydroxide is similar to the pyrolysis temperature of asphalt binder [19,20,21,22]. Layered double hydroxide (LDH) containing magnesium ions, aluminum ions, hydroxyl radicals and carbonate ions have been found to improve the viscosity and high temperature properties of SBS modified asphalt binder, which inherently increases the apparent activation energy and flame retardancy of the asphalt binder [23,24]. The LDH can also be mixed with aluminum hydroxide (ATH) to form synergistic effects that result in achieving better fire resistance properties [1].

Compared with the organic flame retardants, inorganic flame retardants such as mental hydroxides have poor compatibility with asphalt binder. If a good flame retardancy is to be pursued, the dosage of inorganic flame retardants needs to be sufficiently large [4,17,25]. The phase composition of the flame-retardant filler (FR) explored in this study mainly includes magnesium hydroxide, aluminum hydroxide, inorganic phosphate and melamine salt. This type of inorganic FR can directly replace the mineral powder in the asphalt mixture at a relatively large dosage to achieve the effects of good flame retardancy, environmental friendliness and low cost. The influence of the filler-type FR on the processes that it might induce during thermal degradation of FRA have rarely been reported in the literature reviewed by these authors. It is also unclear whether the FR has any effect on the rheological properties or pavement performances of asphalt binders. To evaluate the flammability and multistage pyrolysis characteristics of SBS modified asphalt binders mixed with FR, limiting oxygen index (LOI), smoke density test, TG/DSC test, and thermal kinetics analysis of FRA were conducted. Transformations within asphalt binder in the presence of FR were monitored using Fourier transform infrared spectrometry (FTIR). Likewise, the rheological behaviors of the different asphalt matrix systems were evaluated using the dynamic shear rheometer (DSR) tests. This was necessary to have a fundamental understanding of the rheological properties of FRA in practical applications, especially in the case of a large dosage of FR being used as a filler [19].

Each laboratory test was replicated at six FR dosages ranging from 0 to 50%. From these measurements, the changes in the FRA thermal properties with the variation of FR dosage were comparatively assessed. The effects of filler-type inorganic flame retardant on asphalt binder multistage thermal degradation process and the relevant mode of action were also quantitatively evaluated. The laboratory tests also verified whether the presence of FR influenced the chemical functional groups and rheological properties of the SBS modified asphalt binder. An overall goal is to provide guidance for the use of FRA in tunnel pavements, and to promote an extensive application of an environmental-friendly filler-type flame retardant.

## 2. Materials and Lab Test Methods

### 2.1. Materials

#### 2.1.1. Asphalt Binder

Styrene–butadiene–styrene (SBS) triblock copolymer modified asphalt binder (SBSMA) was obtained from Suzhou Sanchuang Road Engineering Co., Ltd. in Jiangsu province. The dosage of the SBS modifier in SBSMA was 4.5% by weight of the asphalt binder. The SBSMA was tested in accordance with the Chinese specification JTG E20-2011 [26] and its technical indices are listed in Table 1.

#### 2.1.2. Magnesium Hydroxide-Based Inorganic Flame Retardant

A mineral flame-retardant filler for tunnels named Xutai was adopted. It is a magnesium hydroxide-based composite inorganic powder made of a variety of natural minerals which include magnesite, brucite, quartz and calcite. The effective flame retardant components of FR were magnesium hydroxide, aluminum hydroxide, inorganic phosphate, and melamine salt. The technical properties are shown in Table 2.

X-ray fluorescence analysis of the FR revealed its mineral compound composition in the form of oxides, as listed in Table 3. The results further corroborate the phase composition of FR. This type of FR is a filler-type flame retardant. That is, when preparing the samples, it replaces the mineral powder filler in equal amount.

#### 2.1.3. Sample Preparation and Dosages

Flame retardants (FR) were weighed at 10%, 20%, 30%, 40% and 50% of the asphalt binder, respectively, to prepare FRA with various FR dosages. To ensure the homogeneity of distribution of FR in the asphalt mastic, attention was paid to the preparation process. Firstly, the SBS modified asphalt binder was heated up to 175 °C in an electric furnace. Secondly, the FR at a weight percentage of 10~50% was then slowly added into the asphalt binder whilst continuously stirring for 20 min. The stirring process, at a speed of 5000 rpm, was continued for another 30 min to ensure that the FR was homogeneously mixed and blended in the asphalt mastic. Lastly, the prepared FRA samples were then poured into molds, ready for laboratory tests.

### 2.2. Laboratory Test Methods

As discussed subsequently, tests conducted in the study included flame retardancy, synchronous thermal analysis, kinetic thermal analysis, FTIR, and DSR, respectively. For each test, a minimum of 3 sample replicates were tested per test condition per FR dosage.

#### 2.2.1. Flame Retardancy Tests

The LOI test was performed using the FTT 0077 tester (Fire Testing Technology, West Sussex, UK) in accordance with the NB/SH/T 0815-2010 [30] test procedure. The critical oxygen concentration that could sustain the combustion of the asphalt binder was measured using the LOI test. The measured and collected data were expressed as volume percentage of oxygen in the nitrogen-oxygen mixture [31].

Smoke density was evaluated by measuring the loss of the light flux. This reduction in light flux was due to the reflection of light from smoke produced by material combustion. A FTT smoke density tester (Fire Testing Technology, West Sussex, UK) was used on 25 × 25 × 6 mm^3^ sized samples in accordance with the GB/T 8627-2007 test procedure [32]. The smoke density ratings (SDR) of the samples were determined from the generated smoke density curves. Three replicate samples were tested for each FR dosage in the asphalt binder.

#### 2.2.2. Synchronous Thermal Analysis

Thermogravimetric (TG) and differential scanning calorimetry (DSC) tests were carried out simultaneously on pure FR and asphalt binder samples using a synchronous thermal analyzer STA 449 F5 Jupiter (NETZSCH Group, Selb, Germany) to measure the mass loss and thermal effects when subjected to heating. The atmospheric condition used for the thermal analysis test was simulated air, namely a purge gas composed of nitrogen at a flow rate of 40 mL/min and oxygen at a flow rate of 15 mL/min. The protective gas was nitrogen at a flow rate of 20 mL/min. The heating rate has a potential to affect the release rate of gaseous products and the thermal degradation process of asphalt binder during the test [33]. In this study, the temperature was increased from 25 to 800 °C at a constant rate of 10 °C/min. This heating rate was determined through trial and error. A higher heating rate resulted in less resolution between the two adjacent peaks, and some intermediate product information might be lost, whilst a lower heating rate may lead to an unnecessary long test duration and obscure the peak shapes.

The mass of the asphalt binder sample was 8~12 mg. The derivative thermogravimetry (DTG) curves were obtained by differentiating the TG curve, which were subsequently used to analyze the weight loss rate at different combustion stages of the asphalt mastic. Three replicate samples were tested for each FR dosage of the asphalt mastic.

#### 2.2.3. Kinetics of Thermal Analysis

The basis of kinetic analysis revolves around studying the functional relationships between temperature and/or time as independent variables and the degree of reaction as dependent variables. In this study, the Arrhenius model was applied to quantitatively describe the dependence of the reaction rate on temperature, as shown in Equation (1) [34,35].

(1) $$k=Ae^{-\frac{E_{a}}{RT}},$$

where:

*k =* Reaction rate constant at temperature *T*;
*A* = Pre-exponential factor (frequency factor), (s^−1^);
*E*_a_ = Surface activation energy, (J/mol);
*R* = Ideal gas constant, 8.314 (J/mol·K);
*T* = Thermodynamic temperature, (K).

The reaction rate of solid-state reactions under isothermal conditions is described by Equation (2), in which the percent conversion (*α*) could be calculated from the data obtained by thermogravimetric analysis using Equation (3). From Equation (2), the differential form of the reaction rate under non-isothermal conditions (constant heating rate) can be obtained as shown in Equation (4). In Equation (5), the reaction function, *g*(*α*), was defined as an integral form. After separating the variables from Equations (1) and (4), the integral form of the reaction rate under isothermal conditions and constant heating rate were obtained as illustrated in Equation (6) and Equation (7), respectively [36,37,38,39,40].

(2) $$\frac{d\alpha }{dt}=Ae^{-\frac{E_{a}}{RT}}f\left(\alpha \right),$$

(3) $$\alpha =\frac{m_{i}-m_{t}}{m_{i}-m_{f}},$$

(4) $$\frac{d\alpha }{dT}=\frac{d\alpha }{dt}\cdot \frac{dt}{dT}=\frac{d\alpha }{dt}\cdot \frac{1}{\beta }=\frac{A}{\beta }\cdot e^{-\frac{E_{a}}{RT}}f\left(\alpha \right),$$

(5) $$g\left(\alpha \right)=\int_{0}^{\alpha }\frac{1}{f\left(\alpha \right)}d\alpha ,$$

(6) $$g\left(\alpha \right)=k\cdot t=Ae^{-\frac{E_{a}}{RT}}t,$$

(7) $$g\left(\alpha \right)=\frac{A}{\beta }\cdot \int_{0}^{T}e^{-\frac{E_{a}}{RT}}dT,$$

where:

*α* = Percent conversion;
*t* = Reaction time, (s);
d*α*/d*t* = Isothermal reaction rate;
*f*(*α*) = Reaction function in differential form;
*m*_i_ = Initial mass at the beginning of the reaction, (mg);
*m*_t_ = The mass at time *t*, (mg);
*m*_f_ = Final mass after the reaction, (mg);
d*α*/d*T* = Non-isothermal reaction rate;
d*T*/d*t* = Constant heating rate, usually denoted by *β*, (K/s).

The Coats–Redfern integral method was adopted to solve the kinetic parameters for the thermal analysis. Equation (8) is basically the approximate analytical solution [41]:

(8) $$\ln\left[\frac{g\left(\alpha \right)}{T^{2}}\right]\approx \ln\left[\frac{AR}{\beta E_{a}}\left(1-\frac{2RT}{E_{a}}\right)\right]-\frac{E_{a}}{RT},$$

Equation (8) can be transformed into a linear equation (i.e.,Y=aX+b) by neglecting the small value 2*RT*/*E*_a_, where:

(9) $$Y=\ln\left[\frac{g\left(\alpha \right)}{T^{2}}\right],\;X=\frac{1}{T},\;a=-\frac{E_{a}}{R},\;b=\ln\left(\frac{AR}{\beta E_{a}}\right),$$

Then, the least square method was applied to fit the TG data, and the *g*(*α*) with the highest coefficient of determination (*R*^2^) was selected as the reaction function. Based on slope *a* and intercept *b* of the fitting line, the apparent activation energy (*E*_a_) and frequency factor (*A*) of the kinetic parameters for the thermal analysis were calculated using Equation (10) [42] as follows:

(10) $$E_{a}=-aR,\;A=-a\beta e^{b},$$

#### 2.2.4. The Fourier Transform Infrared Spectrometry (FTIR) Test

The FTIR test characterizes the different functional groups and their contents by identifying the absorption spectrum differences of the different groups in tested samples irradiated by an infrared light wave [43,44]. In this study, the Nicolet IS10 FTIR device (Thermo Fisher Scientific Inc., Waltham, MA, USA) was used on the powdered FR samples, with a wave number range of 400~4000 cm^−1^. Thereafter, the obtained time domain interference images were converted into ordinary infrared spectra using Fourier mathematical transformation [45]. Three replicate samples were tested for the pure FR filler.

The ALPHA FTIR spectrometer (Bruker Corporation, Rosenheim, German) was used for the attenuated total reflection-Fourier transform infrared (ATR-FTIR) test on the flame-retarded asphalt binder samples. Multi-functional high-flux ZnSe-ATR crystal was used as the ATR accessory. The test was monitored and controlled using the OPUS software, and the scanning speed and resolution were 24 scan/s and 400~4000 cm^−1^, respectively. The signal-to-noise ratio was 40,000:1, with a resolution of less than 0.8 cm^−1^. Three replicate samples were tested for each FR dosage in the asphalt binder.

#### 2.2.5. The Dynamic Shear Rheological (DSR) Test

To evaluate the influence of FR on the rheological properties of the asphalt binder, a Kinexus dynamic shear rheometer (Malvern Instruments Ltd., Great Malvern, UK) was used for temperature scanning tests on the asphalt binders. A parallel plate configuration with a diameter of 25 mm and a gap width of 1mm was adapted for the high temperature DSR scanning tests. The temperature scanning range was 64 to 94 °C and the temperature incremental interval was 6 °C. The DSR measurements were performed in the strain control mode at a fixed frequency of 10 rad/s, on three replicate samples per FR dosage.

## 3. Test Results, Analysis, and Discussion

### 3.1. Flame Retardancy of FRA

#### 3.1.1. FRA Oxygen Index Results and Analysis

**Figure 1:**
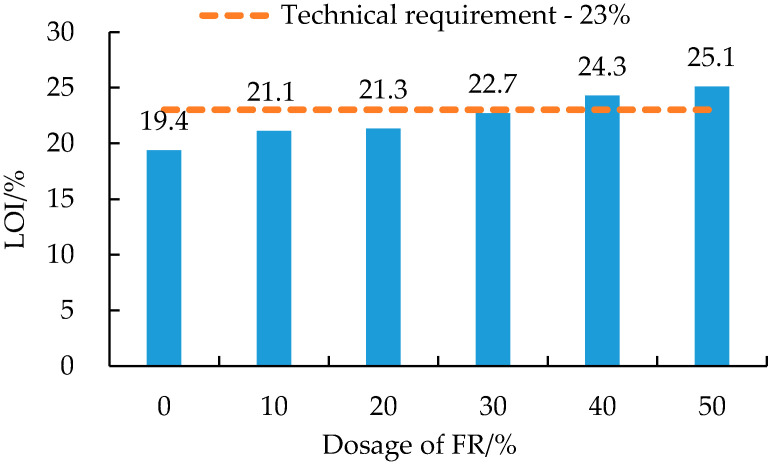
Limiting oxygen index (LOI) results of FRA.

As can be seen from Figure 1, the LOI of FRA increased with an increase in the FR dosage. This variation trend confirmed the obvious effect of magnesium hydroxide-based FR on the flame retardancy of asphalt binder [46]. The LOI value of pure SBS modified asphalt (i.e., 0% FR) was just 19.4, which indicated that the pure asphalt binder had a good flammability in air [1,47]. Compared with pure asphalt binder, the LOI value increased by 9%, 10%, 17%, 25%, and 30%, respectively, after adding 10~50% FR. The LOI of FRA with 40% FR reached 24.3%, meeting the minimum LOI technical requirements (23%) for flame-retarded asphalt in GB/T 29,051 [48]. Therefore, in order to obtain a good flame-retarding performance of FRA, the dosage of FR needs to be higher than 40% by weight of the asphalt binder.

#### 3.1.2. FRA Smoke Density Results and Analysis

**Figure 2:**
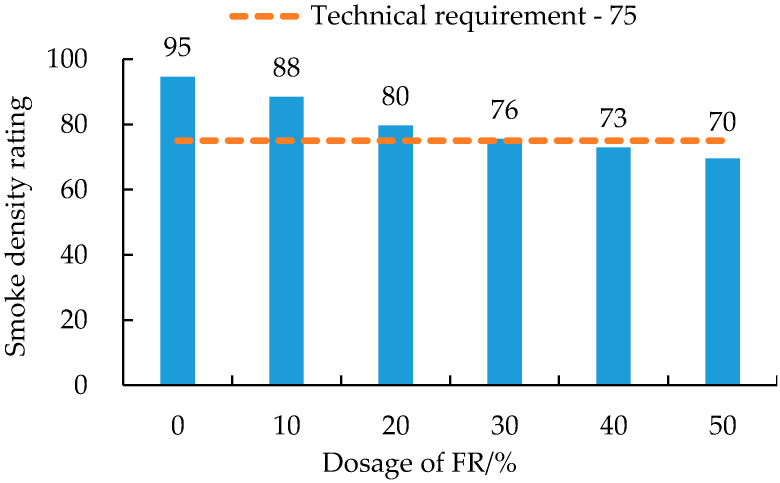
Smoke density rating of FRA.

From Figure 2, the FRA smoke density decreased as the dosage of FR was increased. Compared with the pure asphalt binder (i.e., 0% FR), it decreased by 7%, 16%, 20%, 23% and 27%, respectively, after adding 10~50% FR. At 40% FR, the smoke density was 72.92 and thus, meeting the maximum GB/T 29051 [48] technical requirement of 75. Therefore, to ensure that FRA has the high smoke suppression performance, the amount of FR should be at least 40%. This result is also consistent with the previous findings from LOI test.

### 3.2. Thermal Analysis of FR and FRA

#### 3.2.1. Thermal Degradation Characteristics of FR

**Figure 3:**
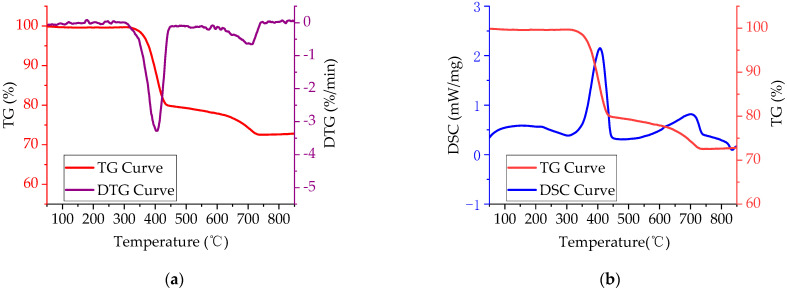
Thermal analysis curves of pure FR: (**a**) TG-DTG curve; (**b**) TG-DSC curve.

From the TG-DTG curve of FR in Figure 3a, there were two mass loss peaks in the heating process of FR. The first stage was 308~451 °C with a peak mass loss rate of −3.28%/min. The second stage was 588~744 °C with a peak mass loss rate of −0.65%/min. The TG-DSC curve for FR in Figure 3b indicates that there existed some corresponding endothermic peaks at both two mass loss peaks, with the peak values of 2.15 mW/mg and 0.81 mW/mg, respectively. Thermal analysis results of FR showed its mode of flame retardancy when the temperature of the FR sample was raised to 308 °C and 588 °C. That is, it began to release crystal water and decomposed, thereby absorbing a large amount of heat, reducing the combustion system temperature, and delaying the burning of the asphalt binder. The decomposition temperature range of the FR matched that of the asphalt binder pyrolysis (the temperature range of the asphalt binder pyrolysis is 245~580 °C), indicating that the FR used in the test could potentially achieve good flame-retarding effects.

#### 3.2.2. Thermal Degradation Characteristics of FRA

**Figure 4:**
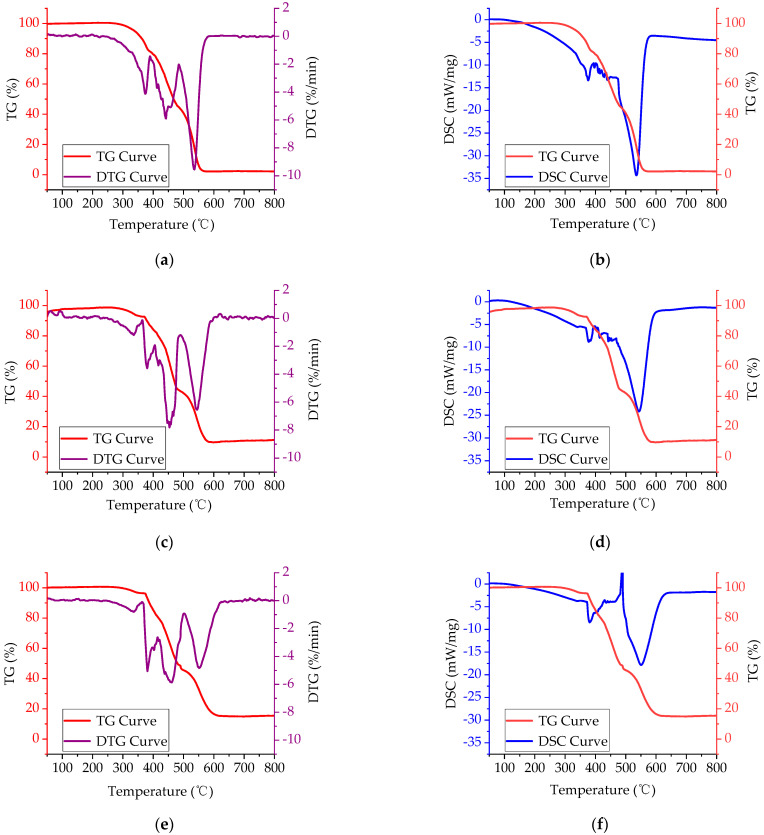

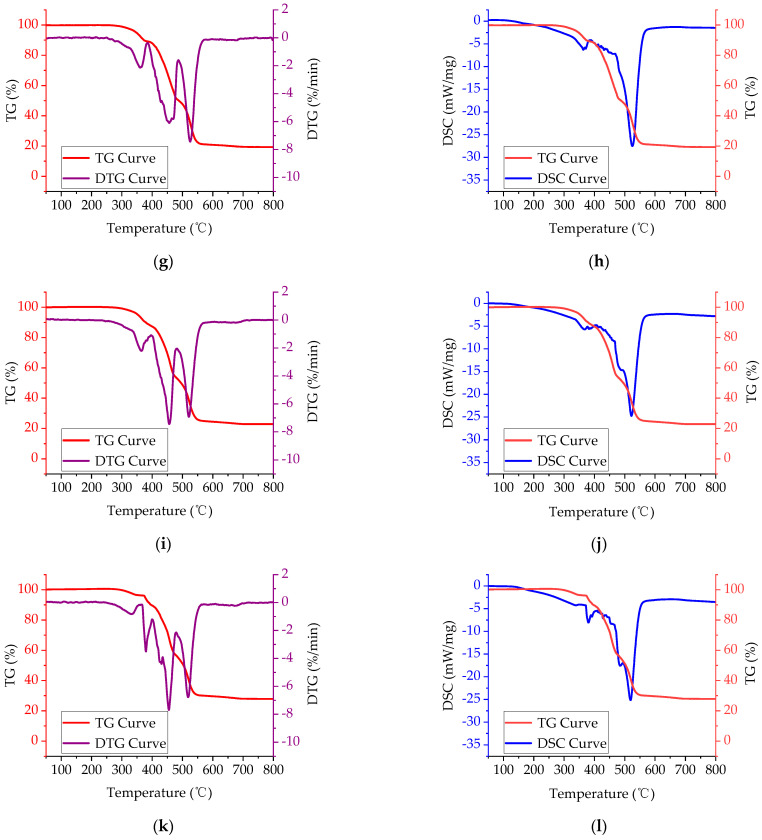
Thermal analysis curves of FRA: (**a**) TG-DTG curve for pure asphalt binder; (**b**) TG-DSC curve for pure asphalt binder; (**c**) TG-DTG curve for 10% FRA; (**d**) TG-DSC curve for 10% FRA; (**e**) TG-DTG curve for 20% FRA; (**f**) TG-DSC curve for 20% FRA; (**g**) TG-DTG curve for 30% FRA; (**h**) TG-DSC curve for 30% FRA; (**i**) TG-DTG curve for 40% FRA; (**j**) TG-DSC curve for 40% FRA; (**k**) TG-DTG curve for 50% FRA; (**l**) TG-DSC curve for 50% FRA.

**Figure 5:**
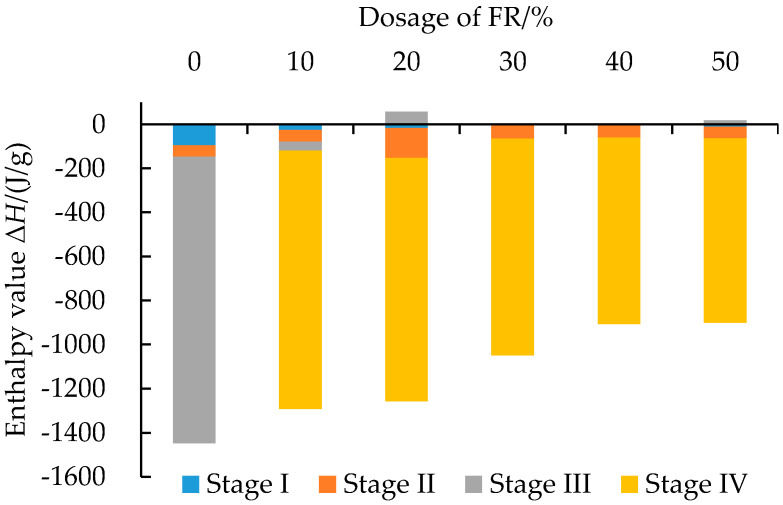
Enthalpy of FRA during the combustion process.

As can be seen from Figure 4, the TG-DSC curves of FRA containing various contents of FR had smaller weight loss rate and lower heat flow peak than that of the pure asphalt binder. This indicated that the addition of FR effectively inhibited the thermal degradation of asphalt binder during the whole test process. Compared with the pure asphalt binder, adding 10~50% FR increased the residue ratio of asphalt binder from 2.1% to 11.6%, 16.1%, 19.3%, 22.8% and 27.9%, respectively. According to the previous study, the residue ratio has been found to have a linear relationship with the LOI value based on a large number of tests, so the higher value of residue ratio could potentially reveal the lower degree of asphalt binder combustion [49]. Furthermore, the peak area of the DSC curve represents the enthalpy value (Δ*H*) of the sample during the thermal analysis test, and the negative enthalpy value indicates the exothermic process [50]. It can be seen from Figure 5 that with an increase in the FR dosage, the total enthalpy of asphalt combustion decreased from −1446.5 J/g to −883.6 J/g and, hence, FR could reduce the total heat release of FRA during the combustion process. All these results suggested that the flame retardancy of asphalt was improved.

To make clear the specific pyrolysis characteristics of the pure asphalt binder and FRA, the thermal analysis curves were divided and analyzed in stages. Based on the mass loss rate, three combustion stages were determined for the pure asphalt binder.

Stage I (245~390 °C): The TG curve gradually decreased, indicating that the light components of the asphalt binder volatilized under heat, and reacted with oxygen to decompose and condense, forming substances such as colloids. The differential scanning calorimetry (DSC) curve had an exothermic peak. In this stage, the C-C bonds and unstable weak bonds of the side chains in the asphalt binder molecules broke and generated radicals, and their interaction with oxygen released heat.

Stage II (390~485 °C): The TG curve decreased significantly, and the width of the pyrolysis peak in the DTG curve was large. This was the main stage of the asphalt binder pyrolysis process. The DSC curve had little change, indicating that the exothermic effects of the asphalt binder were minimal. In this stage, intense pyrolysis occurred in the asphalt binder, namely the light components readily escaped, pyrolysis of the colloids occurred, and thick cyclic aromatic hydrocarbons continued to grow and stacked to each other to generate a large number of asphaltenes and other carbonized products [14].

Stage III (485~580 °C): The TG curve dropped at a higher rate. At this stage, the DTG curve had a pronounced mass loss peak. The DSC curve also had a noticeable exothermic peak, indicating that the asphaltene and other fixed carbon materials started combusting and released a lot of heat. This is the stage when the asphalt binder burned most intensely and degraded most severely.

After FR was added, the first combustion stage of pure asphalt binder was decomposed into the first and second stages of FRA, which led the combustion process to be divided into four stages. The mode of action for FR in each stage were as follows.

Firstly, the magnitude of the pyrolysis peak was diminished in the presence of FR when the temperature was between 260 °C and 350 °C. At this stage, the volatilization of light components in FRA were reduced due to the dilution of the asphalt matrix by the FR filler. The main component of FR had a large heat capacity, which can not only store heat but also conduct heat, so that it is not easy for the asphalt binder to reach the decomposition temperature.

Secondly, the magnitude and width of DTG curve peaks decreased at 350~400 °C. This was the temperature when metal hydroxides in FR started decomposing and producing vapor. The inert gas diluted the concentration of combustible volatile matter in the gaseous phase combustion area on the asphalt binder surface and reduced the temperature of the combustion system, thereby preventing the asphalt binder from intense burning.

Next, in the temperature range of 400~485 °C, both the magnitudes of the mass loss peak in the DTG curve and the exothermic peak in the DSC curve were diminished with the addition of FR. In this stage, the FR continued to decompose rapidly and absorbed a large amount of heat—thus, reducing the exothermic effect and temperature of the FRA system. As a result, the pyrolysis of the colloids, growth of thick cyclic aromatic hydrocarbons and the generation of asphaltenes were inhibited in the asphalt binder.

The last stage ranged from 485 °C to 585 °C. The descent rate of the TG curve, mass loss peak height in the DTG curve, and heat release peak height in the DSC curve decreased considerably in this temperature range. In addition to the effects mentioned above, the active oxides from FR decomposition such as magnesium oxide promoted the charring of the asphalt binder surface, and a tight charring layer has been formed at this stage. The oxide isolation layer covering the asphalt binder surface blocked the contact between oxygen and carbon containing substances in the asphalt binder, resulting in a significant decrease in the flammability of FRA. Thus, the flame-retarding effect was achieved.

#### 3.2.3. Multistage Thermal Degradation Kinetic Analysis of FRA

The combustion reaction function of the asphalt binder was derived using the Coats–Redfern integral method [41], and its parameters for different FRA at different combustion stages were calibrated using the experimental data. The apparent activation energy *E*_a_ and the pre-exponential factor (frequency factor, *A*) were obtained based on the calibrated parameters. Reaction functions selected in this study are shown in Table 4 [51], and the corresponding numerical results are listed in Table 5.

It can be seen from Table 5 that all the coefficients of determination (*R*^2^) of combustion reaction functions for the asphalt binders were greater than 95%. Therefore, the reaction functions could accurately describe the combustion process of the asphalt binders.

The reaction function and apparent activation energy varied in each combustion stage and each FR dosage, respectively. In the first stage, the thermal degradation reaction of the pure asphalt binder mainly followed the D4 (Table 4) mode of action. The pyrolysis reaction rate of the asphalt binder was controlled by the diffusion of the volatile light components to the combustion reaction interface of the asphalt binder surface. The first thermal degradation stage of FRA followed the F1 (Table 4) mode of action, indicating that the existence of FR restricted the nucleation growth and diffusion effects of the asphalt binder pyrolysis.

In the second stage, the combustion reaction of pure asphalt binder followed the R2 (Table 4) mode of action. During the combustion process, the residual products were dehydrogenated and condensed to form polycyclic aromatic hydrocarbons and asphaltenes, as reported in the TG/DSC results. Since it follows the reaction function R2 (Table 4), the decomposition rate of the colloid and other substances was controlled by pushing the nucleation boundary towards the center of the solid particles. The second combustion reaction stage of FRA mainly followed either F1 or F3 modes of action (Table 4), whereas the third stage followed F1 or F2 modes of action (Table 4). In these two stages, the FR apparently diluted the concentration of the combustible components in the gaseous and solid phases of the asphalt binder, thus reducing the combustion reaction rate of the asphalt binder.

**Figure 6:**
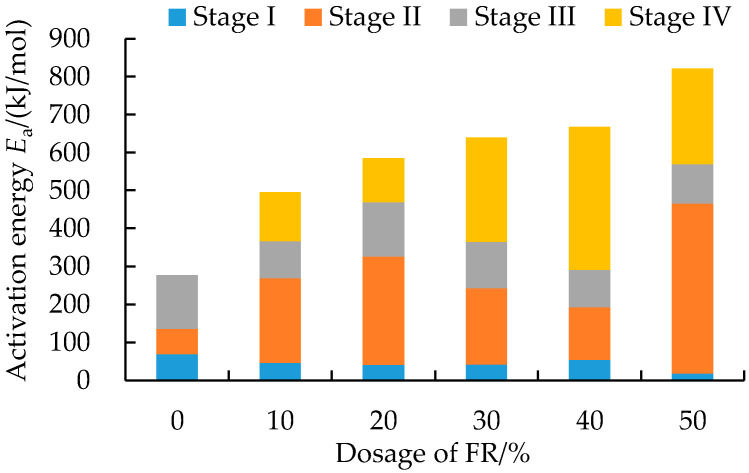
Apparent activation energy of FRA.

In the third stage, the combustion reaction of the pure asphalt binder followed the F1 mode of action (Table 4). According to the reaction function in this stage, the combustion rate of the asphalt binder was proportional to the concentration of reactants and the power index 1 (reaction order = 1) of the reactant residue, which satisfied the first-order model (Mample model [54,55]). The last combustion stage of FRA followed the reaction order model (i.e., the F1, F2 or F3 modes of action in Table 4). The dense oxide protective layer formed by the decomposition of FR during this stage decreased the concentration of the reactants in asphalt binder. The multistage apparent activation energy results of the FRA combustion processes are shown in Figure 6.

It should be noted that the apparent activation energy obtained using the thermal analysis test was a global average value affected by many processes [56]. Different test conditions, such as temperature, may lead to different recorded results. The apparent activation energy shown in Figure 6 was intended to reflect and compare the overall reaction complexity of each type of FRA under the same test condition. From Figure 6, as the dosage of FR increased, the total apparent activation energy of the whole combustion process increased. Compared with the pure asphalt binder, the apparent activation energy for the FRA combustion process increased by 79%, 111%, 131%, 141% and 196%, respectively, with the addition of 10~50% FR, which greatly increased the complexity of the combustion reaction for the asphalt binder. The combustion kinetic analysis of FRA further verified the condensed and gaseous phase flame retardant action mode of FR. FR modification mainly took effect in the second and fourth stages of the FRA combustion process. This did not only delay the combustion of the asphalt binder, but it also reduced the combustion rate, reflecting a good flame-retarding effect.

### 3.3. FTIR Analysis of FRA

**Figure 7:**
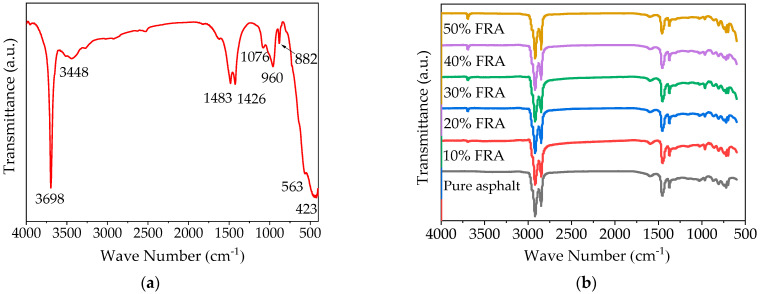
FTIR spectra of FR and FRA: (**a**) FTIR spectra of FR; (**b**) FTIR spectra of FRA.

The FTIR spectra of FR samples are shown in Figure 7a. In the FR functional group region (4000~1330 cm^−1^), the spectral bands at 3698 cm^−1^ and 3448 cm^−1^ were related to the OH stretching vibrations of brucite and water in various states, respectively [57,58]. The absorption bands of the CO antisymmetric stretching vibration of carbonate were observed at 1483 cm^−1^ and 1426 cm^−1^ [58,59]. In the fingerprint region (1330~500 cm^−1^), the peak at 1076 cm^−1^ was anti-symmetric stretching vibration of PO_4_ in the inorganic phosphate, and the 960 cm^−1^ peak was the stretching vibration of P-OH in the inorganic phosphate [60]. The shoulder peak at 563 cm^−1^ was a double degenerate (Eu) mode of the OH oscillating and in-plane translational vibrations. The sharp strong peak at 423 cm^−1^ was a double degenerate (Au) mode of the OH stretching and translational vibrations [61].

As can be seen in Figure 7b, the functional group region in the FTIR spectrum (4000~1330 cm^−1^) for FRA had the same characteristic peaks at 2918 cm^−1^, 2850 cm^−1^, 1599 cm^−1^, 1455 cm^−1^, and 1376cm^−1^ as the pure asphalt binder and the same characteristic peak at 3698cm^−1^ as the FR. The major band at 2918 cm^−1^ and 2850 cm^−1^ was stretching vibration of CH_2_, the 1599 cm^−1^ peak corresponded to C=C phenyl ring skeleton vibration and C=O absorption, the 1455 cm^−1^ peak was related to C-H bond’s in-plane stretching vibration, and 1377 cm^−1^ peak corresponded to shear vibration of CH_3_ bond [62,63]. With an increase in FR dosage, the height of the characteristic peak at 3698 cm^−1^ kept increasing whilst the other peak corresponding to the asphalt binder remained unchanged. The appearance of stronger FR characteristic peaks without generation of new chemical functional groups indicated that the addition of FR to the asphalt binder was basically a physical reaction without any strong chemical reactions [23].

### 3.4. Rheological Behavioral Characterization of FRA

**Figure 8:**
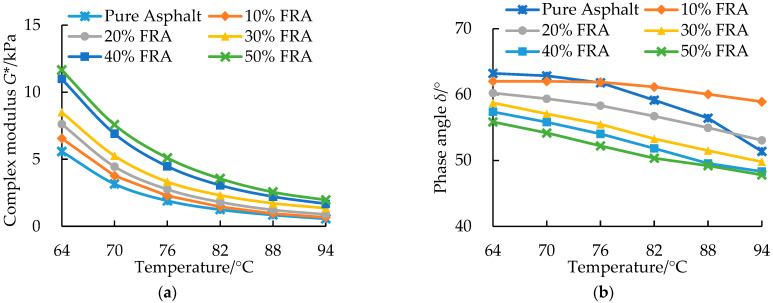
Rheological results and response curves of the pure asphalt binder and FRA: (**a**) complex modulus; (**b**) phase angle.

Figure 8 shows the temperature dependency of the complex modulus (G*) and phase angle (*δ*) of FRA as a function of FR dosage within a temperature of 64~94 °C. From the figure, it is noted that the complex modulus of the pure asphalt binder and flame-retarded asphalt binders decreased exponentially with an increase in temperature. At higher FR dosages, the complex modulus also yielded higher quantitative values. Compared with the pure asphalt binder, the G* value at 64 °C of FRA increased by 108.8% after adding 50% FR. The increase in the G* value was primarily due to the stiffening effect of the added FR particles. As can be seen in Figure 8b, the phase angle of FRA decreased with an increase in the FR dosage. The lower *δ* value, which revealed the more elastic portion of the asphalt binder, resulted from the elasticity provided by the solid particles of metal hydroxide [19].

**Figure 9:**
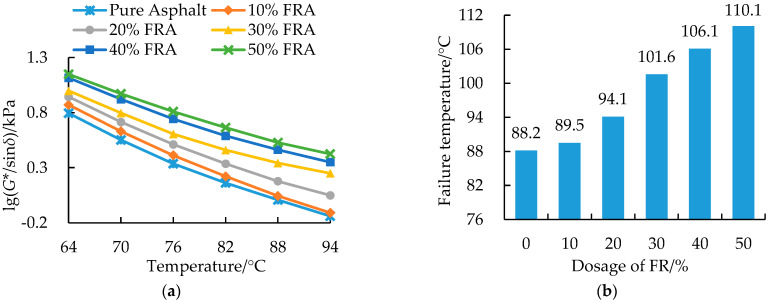
Rutting parameter and critical failure temperature for pure asphalt binder and FRA: (**a**) rutting parameter; (**b**) failure temperature.

The rutting parameter, G*/sin*δ*, is a rheological method to evaluate the rutting resistance of asphalt binders at high temperatures based on the concept of dissipative energy and stress control assumptions. A high rutting parameter represents the high rutting resistance potential and vice versa [64,65]. For asphalt binders, the temperature at which the G*/sin*δ* value equals to 1 kPa is defined as the critical failure temperature [66]. From Figure 9, the rutting parameter for the asphalt binder decreased with an increase in temperature. The curves for G*/sin*δ* and G* value had similar response trends, indicating that the magnitude of the rutting parameter value was mainly affected by the complex modulus (G*). The reason for this was that within the 64~94 °C test temperature region, the variational range of sin*δ* with the phase angle of 50~60 was much smaller than that of the G* value. It can also be seen from Figure 9 that as the FR dosage increased, the critical failure temperature increased as well as the G*/sin*δ* value for the asphalt binder. Adding 50% FR increased the rutting parameter at 82 °C by 218.4%, with a correspondingly increase in the failure temperature by 21.9 °C. Therefore, the addition of FR significantly improved the rheological property of the flame-retarded asphalt binder.

## 4. Conclusions

In this paper, the influence of a flame-retardant filler (FR) on the thermal degradation behavior and rheological characteristic of SBS copolymer asphalt binder was studied. The main conclusions drawn from the study are as follows:

- FR could potentially improve the LOI of FRA and reduce its smoke density. For achieving optimum flame retardancy and smoke suppression performance, the FR dosage should at least be 40% by weight of the asphalt binder when it is used as filler to directly replace mineral powder in tunnel pavement construction;
- FR effectively improved the pyrolysis temperature of the pure asphalt binder, reduced the released heat in the combustion process, and effectively suppressed the flame. The combustion process of the pure asphalt binder and FRA could be divided into three and four stages, respectively. It was apparent from the study findings that FR played the roles of both gaseous and condensed phase flame retardancy. The flame retardant performance was achieved by heat absorption, combustibles dilution, and charring layer formation resulting from FR decomposition;
- Thermal kinetic analysis showed that the combustion reaction of the pure asphalt binder mainly followed the D4, R2, or F1 modes of action, whilst the combustion reaction of FRA primarily followed the reaction order model (F1, F2 or F3 modes of action). The addition of FR significantly improved the apparent activation energy (*E*_a_) of FRA, increased the complexity of the combustion reaction, and improved the flame retardancy performance of the asphalt binder;
- The addition and the variation of the FR dosage did not significantly impact the main functional groups of the asphalt binder according to the FTIR analysis, i.e., it was mainly a physical interaction without strong chemical reactions;
- The complex modulus (G*) value of FRA increased with the addition of FR, whilst the phase angle (*δ*) had an opposite response trend. However, the net effect was that FR improved the rutting resistance of the asphalt binder at high temperatures due to the stiffening effect from its (FR) solid particles.

Overall, the study has indicated that FR is a promising filler material for modifying asphalt binders to enhance their flame retardancy and smoke suppression. However, whilst the study results were plausible, experimentation with different base asphalt binders, comparisons with different filler materials, field validation, and more methods for testing combustion and pyrolysis behaviors are warranted in future studies. Nonetheless, the study enriches the literature through provision of a datum reference for using FR as a potential flame retardant in SBS copolymer asphalt binders.

## Figures and Tables

**Table 1:** Technical indices of SBS modified asphalt binder.

Technical Index		Unit	Test Results	Technical Requirements [26]
Penetration (25 °C, 100 g, 5 s)		0.1 mm	48	40~60
Penetration index (PI)		-	0.7	−0.2~+1.0
Ductility (5 cm/min, 5 °C)		cm	28.6	≥20
Softening point, T_R&B_		°C	89	≥70
Dynamic viscosity (135 °C)		Pa·s	2.81	≤3
Flash point (COC ^1^)		°C	330	≥230
After TFOT ^2^	Mass variation	%	0.14	≤±1.0
	Softening point difference	°C	−4	−12~+10
	Penetration ratio (25 °C)	%	79	≥65
	Ductility (5 °C)	cm	16.2	≥15

^1^ Cleveland Open Cup; ^2^ Thin Film Oven Test.

**Table 2:** Technical indices of the flame retardant.

Technical Index		Test Results	Technical Requirements	Test Methods
Apparent relative density		2.388	-	ASTM C128 [27]
Water content/%		0.8	≤1	ASTM D2216 [28]
Particle size range	<0.6 mm	100	100	ASTM D546 [29]
	<0.15 mm	98.8	90~100	
	<0.075 mm	94.5	75~100	

**Table 3:** FR composition analysis using X-ray fluorescence.

Compound	MgO	SiO_2_	CaO	Al_2_O_3_	Fe_2_O_3_	P_2_O_5_
Content (%)	54.67	5.48	4.07	0.55	0.50	0.11

**Table 4:** Reaction functions in thermal kinetics analysis [51].

Abbreviation	Mode of Action	Integral Form *g*(*α*)	Differential Form *f*(*α*)
A2	Random nucleation and growth, S-shape *α-t* curve, Avrami-Erofeev equation [52]	${\left[-\ln\left(1-\alpha \right)\right]}^{\frac{1}{2}}$	$2\left(1-\alpha \right){\left[-\ln\left(1-\alpha \right)\right]}^{\frac{1}{2}}$
R2	Two-dimensional phase boundary reaction, cylindrical symmetry, area retraction, deceleration *α-t* curve	$1-{\left(1-\alpha \right)}^{\frac{1}{2}}$	$2{\left(1-\alpha \right)}^{\frac{1}{2}}$
D4	Three-dimensional diffusion, spherical symmetry, deceleration *α-t* curve, Ginstling–Brounshtein equation [53]	$1-\frac{2}{3}\alpha -{\left(1-\alpha \right)}^{\frac{2}{3}}$	$\frac{2}{3}{\left[{\left(1-\alpha \right)}^{\frac{1}{3}}-1\right]}^{-1}$
F1	Random nucleation and growth, S-shape *α-t* curve, A1, Mample single rule [54]	−ln(1−α)	1−α
F2	Chemical reaction, deceleration *α-t* curve	1/(1−α)−1	${\left(1-\alpha \right)}^{2}$
F3	Chemical reaction, deceleration *α-t* curve	$1/{\left(1-\alpha \right)}^{2}-1$	${\left(1-\alpha \right)}^{3}$

**Table 5:** Combustion reaction functions and kinetic parameters of FRA.

Type	Stage	Reaction Function Parameters				Kinetics Parameters	
		*a*	*b*	*R* ^2^	Function	*E*_a_ (kJ/mol)	*A* (s^−1^)
Pure asphalt	Stage I	−8341	8.38	0.98	D4	69.3	6.07 × 10^6^
	Stage II	−8042	4.56	0.98	R2	66.9	1.28 × 10^5^
	Stage III	−16,972	19.01	0.99	F1	141.1	5.08 × 10^11^
10% FRA	Stage I	−5564	5.09	0.99	F1	46.3	1.50 × 10^5^
	Stage II	−26,803	58.61	0.99	F3	222.8	1.27 × 10^29^
	Stage III	−11,658	13.19	0.98	F1	96.9	1.04 × 10^9^
	Stage IV	−15,504	15.50	0.98	F1	128.9	1.39 × 10^10^
20% FRA	Stage I	−4917	3.41	0.97	F1	40.9	2.47 × 10^4^
	Stage II	−34,322	76.88	0.95	F3	285.4	1.40 × 10^37^
	Stage III	−17,094	25.46	0.96	F2	142.1	3.33 × 10^14^
	Stage IV	−13,964	11.82	0.96	F1	116.1	3.15 × 10^8^
30% FRA	Stage I	−5041	4.68	0.99	F1	41.9	9.08 × 10^4^
	Stage II	−24,237	53.26	0.98	F1	201.5	5.46 × 10^26^
	Stage III	−14,492	20.54	0.96	F2	120.5	2.02 × 10^12^
	Stage IV	−33,101	50.87	0.98	F2	275.2	6.83 × 10^25^
40% FRA	Stage I	−6511	9.59	0.97	F2	54.1	1.59 × 10^7^
	Stage II	−16,708	35.12	1.00	F3	138.9	4.98 × 10^18^
	Stage III	−11,847	13.92	0.98	F1	98.5	2.20 × 10^9^
	Stage IV	−45,240	76.45	0.98	F3	376.1	1.20 × 10^37^
50% FRA	Stage I	−2176	−4.96	0.99	A2	18.1	2.55
	Stage II	−53,792	129.63	0.96	F3	447.2	1.78 × 10^60^
	Stage III	−12,440	15.30	0.97	F1	103.4	9.11 × 10^9^
	Stage IV	−30,395	46.73	0.98	F2	252.7	9.97 × 10^23^

## Data Availability

Not applicable.
